# Real-Time Monitoring of Pellet Plastication in a Full-Flight Screw and Kneading Disk Elements of a Co-Rotating Self-Wiping Twin-Screw Extruder by Acoustic Emission (AE) Sensing

**DOI:** 10.3390/polym15051140

**Published:** 2023-02-24

**Authors:** Tsukasa Kida, Masatoshi Ohara, Keigo Inamori, Shogo Nagasawa, Shin-ichi Kihara, Kentaro Taki

**Affiliations:** 1 Department of Natural System, Graduate School of Natural Science, Kanazawa University, Kanazawa 920-1192, Japan; 2 Shibaura Machine, Numazu 410-8510, Japan; 3 Graduate School of Advanced Science and Engineering, Hiroshima University, Higashihiroshima 739-8527, Japan; 4 School of Frontier Engineering, Kanazawa University, Kanazawa 920-1192, Japan

**Keywords:** pellet plastication, acoustic emission, melt removal, twin-screw extrusion

## Abstract

The plastication of pellets in a co-rotating twin-screw extruder is a significant concern for product homogeneity and stability in the plastic industry. We developed a sensing technology for pellet plastication in a plastication and melting zone in a self-wiping co-rotating twin-screw extruder. The collapse of the solid part of the pellets emits an elastic wave as an acoustic emission (AE) that is measured on the kneading section of the twin-screw extruder using homo polypropylene pellets. The recorded power of the AE signal was used as an indicator of the molten volume fraction (MVF) in the range of zero (fully solid) to unity (fully melted). MVF decreased with increasing feed rate monotonically in the range of 2–9 kg/h at a screw rotation speed of 150 rotations per minute (rpm) because of the reduction in the residence time of pellets in the extruder. However, the increase in feed rate from 9 to 23 kg/h at 150 rpm resulted in an increase in the MVF as the friction and compaction of pellets caused their melting. The AE sensor could elucidate the pellet’s plastication phenomena caused by friction, compaction of pellets, and melt removal in the twin-screw extruder.

## 1. Introduction

Co-rotating self-wiping twin-screw extruder is a continuous polymer processing machine and one of the most widely used screw extruders in the polymer processing industry. It produces pellets, sheets, films, and other plastic products efficiently. The twin-screw extruder transports, plasticates, and melts pellets of plastic resin. When pellets of thermoplastic resin are fed to the twin-screw extruder, the heat transfer from the barrel of the extruder, friction, and adiabatic compression of pellets result in plastication and melting of pellets [1]. If the pellets do not plasticate well in the plastication zone, mixing with additives in the subsequent compounding zone becomes incomplete. Plastication and melting in polymer processing machines are critical elementary steps because they are often the rate-controlling steps that consume 70–80% of the total processing energy input [1]. Additionally, during the melting of polymer blends, a major part of the blend morphology is established [1].

The plastication and melting of pellets in a co-rotating twin-screw extruder have been studied mainly by building mathematical models and performing numerical simulations of the plastication zone over the past three decades [2,3,4]. These models assume that melting occurs primarily by the viscous energy dissipation during the flow of suspensions of solid polymer particulates in melts, with the evolution of melting decreasing the particulate size [1]. Other studies discussed plastic energy deformation via compressive experiments on molded disks of several materials [5,6,7]. Zhu et al. investigated single pellet deformation using finite element analysis and reported that mechanical energy is converted to heat in this process [8]. The mathematical models and numerical simulations were evaluated by the limited experimental results. Despite the extensive research, studies on real-time monitoring in the plastication zone are limited [9].

Real-time monitoring can raise the alarm and eventually stop the line when the pellet plastication is insufficient. It readily provides the state of pellet plastication and helps understand the phenomenon. However, the resin temperature and pressure sensors are too fragile to be used in the solid pellets and plastication zones as the solid pellets may collide with the sensors and damage them. Hence, a new type of sensor for resin plastication monitoring is demanded. 

In this study, we focused on acoustic emission (AE). AE originates from the transient stress waves that are generated by crack growths and many other kinds of material degradation and deterioration [10]. AE can be applied for monitoring mechanical behaviors of various materials: carbon/epoxy composites [11], glass fiber reinforced polypropylene [12], high-density polyethylene/polypropylene blends [13], natural fiber composites [14], carbon fiber reinforced plastic [15], degraded polypropylene [16], polypropylene/cement [17], etc. Recently, machine learning technology has been applied to the pattern recognition of the AE signal to determine the extent of the damage [18,19].

According to the previous studies on applying AE sensing to various materials, we can expect that collapsing resin pellets in the kneading screw elements emit the AE signal in the extruder.

The AE sensor comprises a piezoelectric transducer and detects ultrasound waves as the AE signal. The ultrasonic wave receiver and transmitter have been applied to the extruders in polymer processing to evaluate the density of polymer melt and the composition of polymer blends [20]. Previous studies have measured the density of molten resin that can be applied to the analysis of plastication in the twin-screw extruder. However, the change in density cannot be directly explained by the change in plastication. The AE signal depends on physical incidents, such as elastic deformation, destruction, and failure of materials, which are directly related to pellet plastication. 

Here, a measurement technique to monitor pellet plastication in the extruder using an AE sensor was developed. The effects of screw speed, flow rate, and plastication of polypropylene pellets in the twin-screw extruder were investigated. The experimentally obtained data were discussed with the suggested mechanism previously reported by Gogos et al. [1].

## 2. Materials and Methods

### 2.1. Materials

A homo polypropylene (PP, F-704NP) with a melt flow rate (MFR) of 7.0 g/10-min was acquired from Prime Polymer, Minato City, Tokyo, Japan. The resin was used as received. The parameters of the Cross model fitted to the complex viscosity are shown in the Appendix A. The melting temperature was measured using a differential scanning calorimeter (DSC-8500, PerkinElmer, Waltham, MA, USA) with a heating rate of 10 °C/min under 20 mL/min flow of nitrogen. The DSC was calibrated with the Indium standard. The melting temperature was 164 °C.

### 2.2. AE Sensor

An AE sensor (FAEN-S601, FIRST AE), a built-in amplifier, a 2nd amplifier (EDGE NODE DISCOVERY SEG), and a data acquisition A/D converter (NI DAQ cDAQ-9171 and NI-9775, National Instruments) were connected sequentially. In-house LabVIEW^®^ software was used to collect and record the AE data. The data acquisition speed was 250 kHz, and the sampling period was 0.2 s.

**Figure 1:**
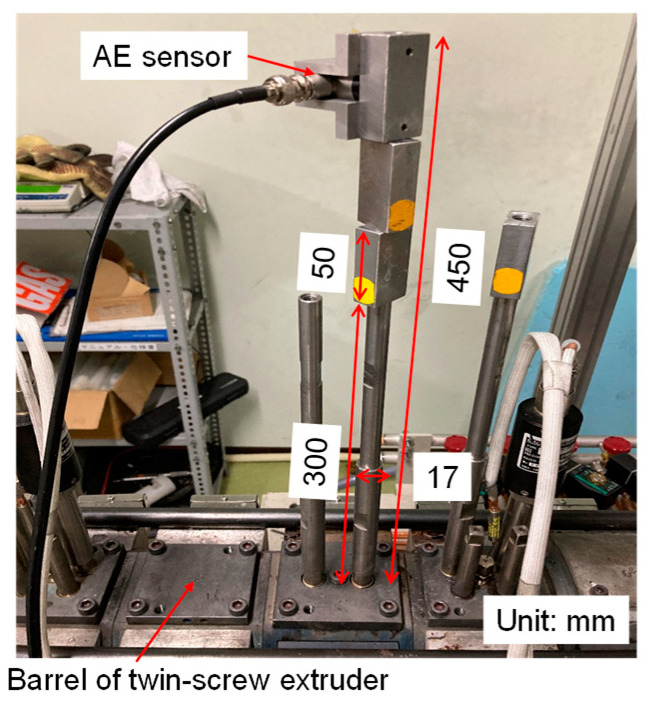
Image of the AE sensor, waveguide, and barrel of the twin-screw extruder.

The AE sensor was attached to a waveguide with a diameter of 17 mm and a length of 300 mm screwed on the barrel of the twin-screw extruder, as shown in Figure 1, to avoid any heat damage to the AE sensor. Three metal blocks, 50 mm in length, were attached to the waveguide. The distance between the barrel surface and the AE sensor was 450 mm. A certain amount of grease (EchoZ+, ECHO ultrasonics) was applied between the AE sensor and the waveguide to reduce the transmission loss. To attach the AE sensor securely to the waveguide without damaging the AE sensor, the same fixing torque (0.35 N∙m) should be maintained for every measurement when the AE sensor was fixed on the waveguide. A wind fan was used to continuously cool the sensor.

### 2.3. Screw Configuration

**Figure 2:**
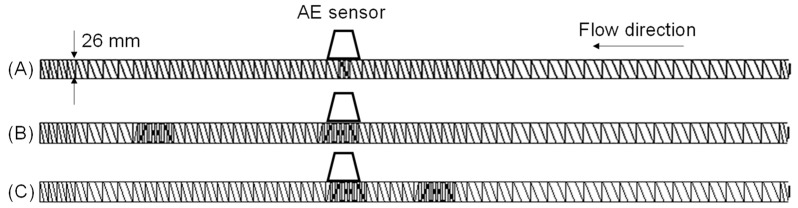
Screw configurations of (**A**–**C**) of the co-rotating twin-screw extruder.

A co-rotating self-wiping twin-screw extruder (nominal screw diameter 26 mm indicated in Figure 2, screw length to diameter ratio, L/D is 64, TEM-26SX) was used. The screw consists of various screw elements, i.e., a piece of the screw. The order of the screw element can be changed to build a desired screw configuration. We used three different screw configurations (A), (B), and (C) to study the plastication in the twin-screw extruder. The order of screw element’s code of screw configurations in the SHIBAURA MACHINE catalog is available in the Appendix A.

Configuration (A): It is a simple screw configuration in which one kneading disk element is at 977.5 mm from the feed, and the others are full-flight screw elements, as shown in Figure 2A. The AE sensor was attached above the five-disk forward kneading element. A barrel temperature of 30 °C was used to identify the AE signal of collapsing solid pellets, while that of 195 °C with a short distance of screw heated at 150 °C immediately downstream of the feed port was used to confirm that the molten resin does not emit the AE signal. It should be noted that the barrel temperature of 195 °C was higher than the melting temperature of the sample PP. The examined screw rotation speed and feed rate were 50 screw rotations per minute (rpm) and 2.0 kg/h, respectively.

Configuration (B): The screw configuration (B) was built to understand the effects of feed rate and screw speed on the plastication phenomena in a full-flight screw, as shown in Figure 2B. The five-disk forward, neutral, and backward kneading elements of 27 mm in length located at 965 mm from the feeding port collapse the partially solid pellets that emit the AE signal. The AE signal was used to understand pellet plastication in upstream full-flight screw elements. The barrel temperature was 195 °C at regions away from four full-flight screw elements in front of the feed port. Thus, the melting zone was located at 965 mm from the feeding port. The screw rotation speed increased as follows: 30, 50, 100, 125, 150, 200, and 250 rpm. The feed rate increased as follows: 2.0, 4.0, 6.0, 12, 18, and 23 kg/h. The interval of the measurement was approximately 3 min. Measurements of 12 kg/h at 30 rpm and 18 and 23 kg/h at 30 and 50 rpm were not performed because the pellets were not fed to the feed port continuously.

Two series of experiments were performed. In the 1st series, the screw rotation speed was increased while the feed rate was maintained constant. Subsequently, the process was examined at a higher feed rate from low to high screw rotation speed. In the 2nd series, the feed rate was increased while the screw rotation speed was maintained constant.

In addition, to collect the AE signal of fully solid pellets for the normalization of the AE signal, the barrel temperature was set equal to the melting temperature at 164 °C.

Configuration (C): The screw configuration (C) was considered to understand the effect of kneading disk elements on the plastication phenomena, as shown in Figure 2C. The five-disk forward, neutral, and backward kneading elements were replaced with full-flight screws. The pellets were plasticized in the replaced kneading disk zone. The AE signal was measured at the downstream kneading zone. The AE signal is possibly generated in the upstream and downstream kneading zones. As the distance between them is set sufficiently long, the AE sensor does not interfere with the AE signal from the upstream kneading zone. 

### 2.4. Residence Time Measurement

The mean residence time of the solid pellets on the full-flight screw was measured as follows. The screw configuration was as same as the upstream configuration from the AE sensor of (B) in Figure 2. The detailed configuration is shown in Table A2 in the Appendix A.

The barrel temperature was set to a room temperature of 30 °C. The pellets were fed to the rotating twin screws alone, consisting of full-flight screw elements and no kneading disk elements. The exit of the extruder was open, and no die was attached to it. This operation does not collapse and melt the pellets. The pellets were only transported to the exit of the extruder by the rotation of screws.

In the measurement operation, the rotating screws and the feeder of pellets were abruptly stopped when the mass flow rate reached a steady state of the desired value. The screws were rotated again to transport the remaining pellets in the barrel when the feeder stopped. All the pellets in the barrel were collected, and the mass (*m*_p_) was measured. The mean residence time per meter was calculated as follows:

(1) $$\bar{\tau }=\frac{m_{p}}{Q_{m}L_{m}}$$

where *m*_p_ (kg) is the remained mass of pellets in the barrel when the screws stopped, *Q*_m_ (kg/s) is the pellets’ mass flow rate in the feeder, and *L*_m_ (m) is the measured barrel distance, which is 0.918 m from feed to head.

The screw rotation speed was changed from 50 to 250 rpm with 50 rpm intervals. The feed rate was increased as follows: 2, 4, 6, 9, 12, 18, and 23 kg/h. The residence time per meter ranges from 9.8–76.5 s/m. The results are summarized in Table 1, and the dependences on the screw rotation speed and the feed rate are plotted.

### 2.5. Signal Processing

Extrusion of pellets at a barrel temperature of 30 °C produced collapsed pellets in the screw configuration (A) at the screw rotation speed and feed rate of 50 rpm and 2.0 kg/h, respectively. The pellets collapsed between the barrel and screws. The pellets were fully melted, and a clear molten strand was obtained at the exit of the die at the barrel temperature of 195 °C at the same screw rotation speed and feed rates at 30 °C.

**Figure 3:**
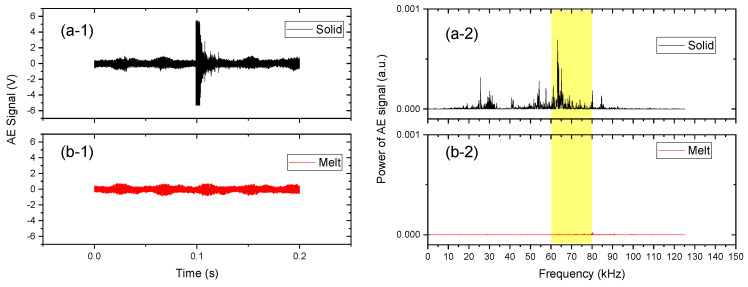
Time domain AE signals at a barrel temperature of (**a-1**) 30 °C and (**b-1**) 195 °C. Power of AE signals in frequency domains at (**a-2**) 30 °C and (**b-2**) 195 °C. The screw rotation speed and feed rate were 50 rpm and 2.0 kg/h, respectively.

A typical time domain and the fast Fourier-transformed AE signals obtained at the barrel temperatures of 30 °C and 195 °C are shown in Figure 3. A strong signal was captured at 0.1 s at 30 °C while no distinct signal was observed at 195 °C in the full range of the time-space. The frequency domain signal indicates that the peaks in the range of 60–80 kHz originated from the AE signals of the collapsing pellets. The signals in the range of 20–60 kHz were superimposed with those of the other phenomena, such as the open-and-close solenoid valves, metal contacting screw and barrel, and other un-clarified noises. Thus, the signals in 60–80 kHz were used in this study as the AE signal of collapsing pellets.

### 2.6. Molten Volume Fraction (MVF) from AE

**Figure 4:**
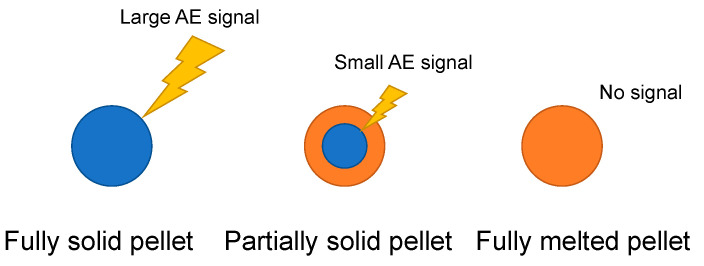
Model of AE signal generation.

A model of AE signal generation, as illustrated in Figure 4, indicates that the AE signal is proportional to the residual solid part of pellets. The recorded time domain signal was processed by the 60–80 kHz bandpass filter. The square of the time domain signal was accumulated during the measurement time of 210 s. Then, the accumulated signal was divided by the number of pellets passed through under the AE sensor for 210 s, which can be calculated by the mass flow rate, measurement time, and average mass of one piece of pellet.

Finally, the MVF, *χ*_p,_ is calculated as follows:

(2) $$\chi_{p}=1-\frac{S\left(T_{b},Q,N_{S}\right)}{S\left(T_{m},2\;\mathrm{kg}/h,\;250\;\mathrm{rpm}\right)}$$

where *S*(*T*_b_, *Q*, *N*_s_) is the accumulated signal per pellet. *S*(*T*_m_, 2 kg/h, 250 rpm) is the reference signal. We could not use the data at room temperature, i.e., at 30 °C, because the pellets could not pass through the three kneading disks, and the motor’s torque increased the specification value. The pellets extruded at *T*_m_ (164 °C), 2 kg/h, and 250 rpm seemed almost solid and did not stagnate in the kneading disk elements. The further increase in feed rate and/or screw rotation speed caused the torque-over. When the only kneading disk, such as in the case of screw configuration (A), was used, the torque-over did not occur. However, some solid pellets passed through the one-kneading disk zone without collapsing. Hence, we placed three kneading disks to ensure all the pellets collapsed in the kneading zone.

## 3. Results and Discussion

### 3.1. Visual Observation of Partially Molten State Pellets on Rotating Screws

**Figure 5:**
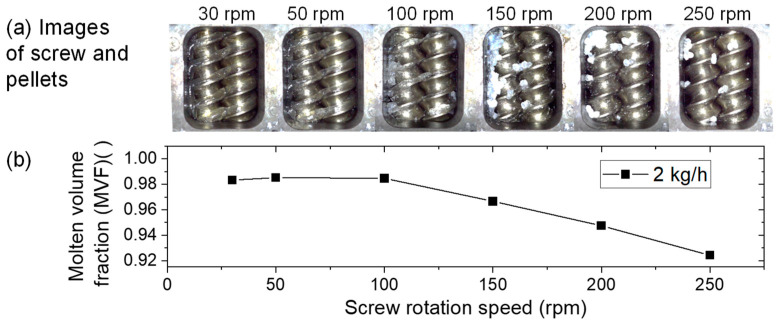
Effect of screw rotation speed on the molten volume fraction (MVF). (**a**) Visual observation and (**b**) MVF. The screw configuration was (B). The feed rate was 2 kg/h. The barrel temperature was 195 °C. The videos of the visual observations are available in the Supplementary Materials.

Figure 5a shows the effect of screw rotation speed on pellet plastication. The full-flight screws in an open barrel immediately upstream of the kneading disk under the AE sensor were observed. The characteristic aspects of melting resin varied with the screw rotation speed. The structural aspects of melting pellets in a co-rotating twin-screw extruder were described by Gogos et al. [21,22,23]. They found seven different states and described their characteristics and definitions. The observed structural state was mentioned following their definition in the parentheses. The clear molten resin on the pushing side of the full-flight screw was observed at 30 and 50 rpm (melt film). The partially melted pellets and clear molten resin were observed at 100 rpm (melt-rich suspension). The partially molten white pellets and their blocks were observed at 150 and 200 rpm (clustered structures). Almost solid pellets rolling on the flight screws were observed at 250 rpm (individual particles).

Figure 5b shows the MVF for each screw rotation speed. The MVF decreases with increasing screw rotation speed. Compared with the images in Figure 5a, the dropping of MVF corresponds to the solid pellet appearance clearly beyond 100 rpm. The MVF can be used to evaluate pellet plastication quantitatively.

### 3.2. Effect of Increase in Screw Rotation Speed on Plastication at a Constant Feed Rate

**Figure 6:**
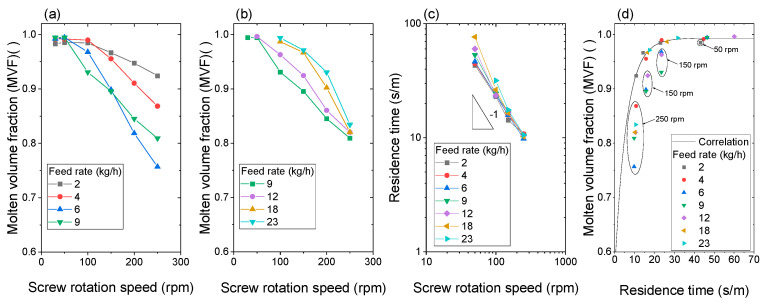
Effect of increase in the screw rotation speed on the molten volume fraction (MVF). The feed rate of (**a**) 2–9 kg/h, (**b**) 9–23 kg/h, and (**c**) residence time per meter of solid pellets in full-flight screws. The residence time was measured for the solid pellets at 30 °C. (**d**) Relationship between the residence time and molten volume fraction (MVF). The screw configuration was (B).

Table 2 and Figure 6a,b show the effect of screw rotation speed on the MVF at a constant feed rate. The MVF for every feed rate decreased with the screw rotation speed. Figure 6c shows the residence time per meter of solid pellets estimated by Equation (1). The residence time per meter monotonically decreased with increasing screw rotation speed. The slopes of the low feed rate are close to −1, indicating that the residence time per meter is proportional to the reciprocal of screw rotation speed. The majority of pellets were transported by the drag force of flight screws. The plots of high feed rate, e.g., 12, 18, and 23 kg/h, in slow-screw rotation speed, deviate from the slope of −1 because the holdup of pellets occurred at the high feed rate and slow-screw rotational speed. 

Figure 6d shows the relationship between the MVF and residence time per meter. Overall, the long residence time resulted in high MVF. Therefore, solid pellets must receive heat from the barrel and screws to melt. Hence, the longer the residence time per meter (more than 25–30 s/m in the polymer), the more heat the pellets received, and pellet plastication advanced. However, there are several plots enclosed by circles that deviate from the overall trends. To segregate them, an empirical equation, Equation (3) was fitted to the plots except for the enclosed ones:

(3) $$\alpha =A_{1}\exp\left(-x/t_{1}\right)+y_{0}$$

where the parameters of Equation (3) were determined by fitting to the experimental data except the data plots enclosed by circles. *A*_1_ = −0.434, *t*_1_ = 5.79239 (s/m), *y*_0_ = 0.99309. The plots on the correlation line and deviated plots indicate that our definition of MVF of Equation (2) is less sensitive to lower MVF and high solid content. Further study is required for the appropriate definition of MVF. 

### 3.3. Effect of Increase in Feed Rate on Plastication at Constant Screw Speed

**Figure 7:**
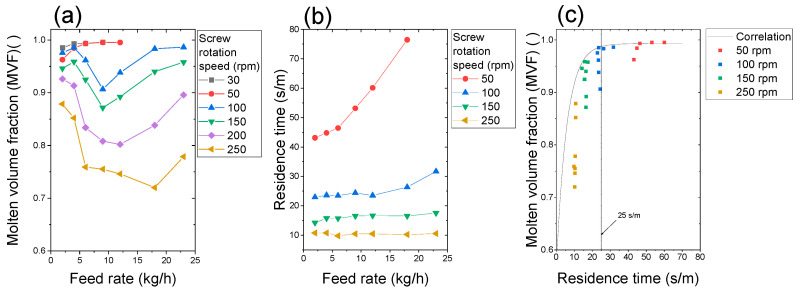
Effect of feed rate on (**a**) the molten volume fraction (MVF) and (**b**) the residence time of pellets. The residence time was measured for the solid pellets at 30 °C. The screw configuration was (B). (**c**) Relationship between the MVF and the residence time per meter. The correlation line was drawn using the parameters and Equation (3).

Table 3 and Figure 7a show the effect of feed rate on the MVF at constant screw rotation speed. The MVF decreased at a lower feed rate and turned to increase at higher feed rates for a screw rotation speed higher than 100 rpm. A minimum MVF for each screw rotation speed existed. The feed rate of the minimum MVF shifted to a high feed rate with increasing screw rotation speed. MVF increased monotonically for the cases of 30 and 50 rpm. 

Figure 7b shows the effect of feed rate on the residence time per meter. The residence time per meter of each screw rotation speed against the feed rate had no peak. The residence time per meter increased with the feed rate, and its slope depended on the screw rotation speed. A monotonical increase in the residence time per meter above 25–30 s/m anticipates the advance of pellet plastication. 

Interestingly, the MVF curves at 50 rpm and those at 100 rpm are different in Figure 7a. The MVF at 100 rpm dropped significantly at 5 and 7 kg/h. The pellets received substantial heat from the barrel at 30 and 50 rpm due to the long residence time. At more than 100 rpm, the residence time was not sufficient to melt the crystalline phase of polypropylene. Thus, the MVF dropped with an increase in the feed rate. Semicrystalline polymer requires heat to reduce the viscosity of the amorphous phase as well as to melt the crystalline phase.

Figure 7c shows the relationship between the MVF and the residence time per meter. The correlation line was drawn by the parameters of Equation (3). The MVF lower than 25 s/m deviated from the correlation line. It indicates that additional shear heating occurred in the starved situation by screw rotation and increased the MVF gradually.

**Figure 8:**
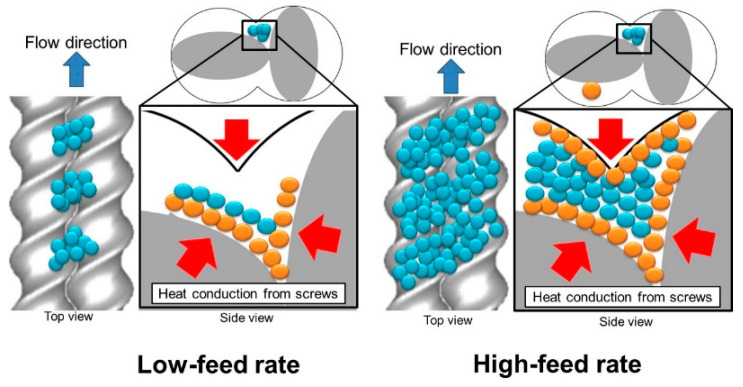
Comparison of the accumulation of pellets in the valley of twin screws in the full-flight screw zone between low feed and high feed rates at a constant screw rotation.

Figure 8 illustrates the accumulation of pellets in the valley of the full-fight screw zone of twin screws. An increase in the feed rate accumulates the pellets between the barrel and screws. The heat conduction rate of the blue-colored pellets in the side view is lower than that of the orange-colored pellets because the heat conducts through the orange-colored pellets. The amount of received heat per pellet at a high feed rate is lower than that at a low feed rate. Thus, the decrease in MVF was caused by the decrease in the amount of heat per pellet with the increase in feed rate. 

**Figure 9:**
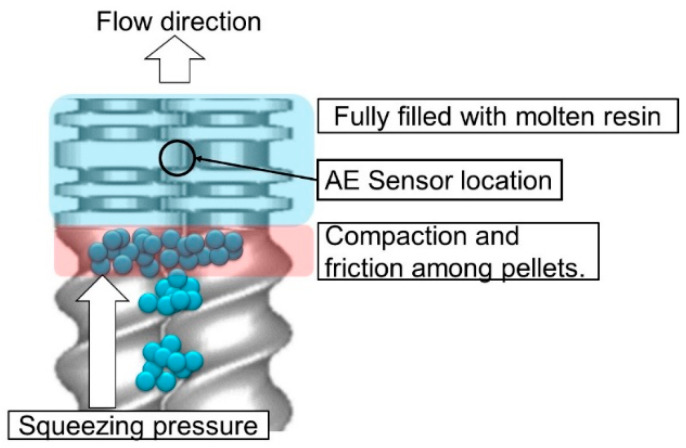
Holdup of pellets by the kneading disk in the twin-screw extruder.

Figure 9 illustrates the holdup of pellets by the kneading disk just below the AE sensor in twin screws. The increase in feed rate causes the holdup of pellets just in the zone before the kneading disk element. The friction and compaction of pellets produce heat, which melts themselves in the zone [23]. Thus, the higher feed rate increased the holdup, friction, and compaction of pellets in the zone. It melts and transforms pellets to increase the MVF at further high feed rates.

### 3.4. Effect of Kneading Disk

**Figure 10:**
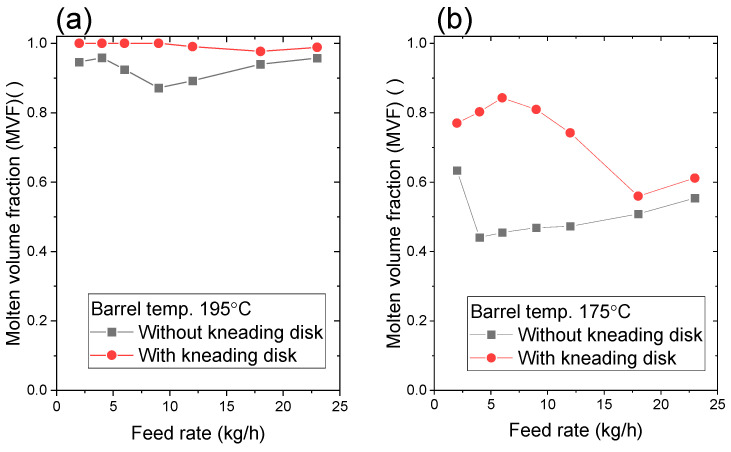
Effect of kneading disk on the molten volume fraction (MVF). The screw rotation speed is 150 rpm. The barrel temperatures were (**a**) 195 °C and (**b**) 175 °C, respectively. The screw configuration of “with kneading disk” was (C) and that of “without kneading disk” was (B).

The effect of the kneading disk was investigated by replacing the full-flight screw with the kneading disk (configuration (C)). Figure 10a shows that the MVF with the kneading disk is 1.0–9.0 kg/h. Further increase in feed rate slightly decreased the MVF to 0.98. The MVF without the kneading disk (configuration (A)) was lower than that with a kneading disk. The kneading disk facilitated the plastication of pellets efficiently. Figure 10b shows the MVF at a barrel temperature of 175 °C. As expected, the MVF drastically decreases and changes nonlinearly with the feed rate, as observed in Figure 7a. The kneading disk does not always plasticate pellets completely.

## 4. Conclusions

Plastication of semicrystalline resin pellets in a twin-screw extruder was investigated using the newly developed AE sensing system, which detects the elastic wave and prevents the collapse of the partially melted pellets in the twin extruder. The MVF based on the power of the AE signal per pellet was defined. The feed rate and screw rotation speed affected the MVF nonlinearly. The residence time, accumulation of pellets, friction between pellets, and compaction play significant roles in plastication. Their contribution changes according to the feed rate. Moreover, the kneading disk efficiently enhanced the plastication independent of the feed rate. The compaction of pellets in the kneading disk zone is a significant factor in plastication.

Measurement of plastication using AE sensing has several advantages. The AE sensor does not contact an object directly. The AE sensing can be applied to severe situations in pressure transducers and will detect abnormal plastication via a sudden increase in the AE signal. 

The AE sensing and investigation of plastication still have some limitations. Our AE sensing data support the previously proposed plastication mechanism. However, further quantitative analysis with mathematical models, such as finite and discrete element methods, is required. Moreover, plastication is followed by mixing additives, resin, and glass fibers in a typical extrusion process. Therefore, it is necessary to identify and categorize problems arising from mixed signals of glass fiber breakage, inorganic particles, and the blending of different viscoelastic materials.

## Figures and Tables

**Table 1:** Residence time per unit meter of solid pellets transported on the full-flight screws at 30 °C.

Residence Time per Meter (s/m)		Screw Rotation Speed (rpm)			
		50	100	150	250
Feed rate (kg/h)	2	43.1	22.9	14.3	10.8
	4	44.8	23.6	15.8	10.8
	6	46.5	23.5	15.7	9.8
	9	53.2	24.4	16.6	10.5
	12	60.1	23.5	16.7	10.5
	18	76.5	26.4	16.6	10.2
	23	- *	31.7	17.6	10.6

* Feed neck occurred. Screw configuration was the “Residence time meas.” in Table A2.

**Table 2:** Summary of molten volume fraction (MVF) dependence on increasing screw rotation speed at constant feed rates: 1st series *.

MVF ( )		Feed Rate (kg/h)						
		2	4	6	9	12	18	23
Screw rotation speed (rpm)	30	0.98	0.99	0.99	0.99	-	-	-
	50	0.99	0.99	0.99	0.99	1.00	-	-
	100	0.98	0.99	0.97	0.93	0.96	0.99	0.99
	150	0.97	0.96	0.90	0.90	0.92	0.97	0.97
	200	0.95	0.91	0.82	0.85	0.86	0.90	0.93
	250	0.92	0.87	0.76	0.81	0.82	0.82	0.83

* Screw configuration was (B).

**Table 3:** Summary of molten volume fraction (MVF) dependence on increasing feed rate at a constant screw rotation speed: 2nd series *.

MVF ( )		Feed Rate (kg/h)						
		2	4	6	9	12	18	23
Screw rotation speed (rpm)	30	0.99	0.99	0.99	1.00	-	-	-
	50	0.96	0.98	0.99	1.00	1.00	-	-
	100	0.98	0.99	0.96	0.91	0.94	0.98	0.99
	150	0.95	0.96	0.92	0.87	0.89	0.94	0.96
	200	0.93	0.91	0.83	0.81	0.80	0.84	0.90
	250	0.88	0.85	0.76	0.76	0.75	0.72	0.78

* Resin is PP-MMFR, and the screw configuration is (B).

## Data Availability

Data are available in a publicly accessible repository.

## Supplementary Materials

The following supporting information can be downloaded at: https://www.mdpi.com/article/10.3390/polym15051140/s1; Video S1: 2kgh100rpm.mp4, Video S2: 2kgh150rpm.mp4, Video S3: 2kgh200rpm.mp4, Video S4: 2kgh250rpm.mp4, Video S5: 2kgh30rpm.mp4, Video S6: 2kgh50rpm.mp4.

## Appendix A

The equations of the temperature-dependent Cross model are shown in Equations (A1) and (A2):

(A1) $$\eta =\frac{\eta_{0}}{1+{\left(\frac{\eta_{0}\dot{\gamma }}{\tau^{*}}\right)}^{\left(1-n\right)}}$$

(A2) $$\eta_{0}=B\exp\left(\frac{T_{b}}{T_{r}}\right)$$

where *η* is the temperature-dependent viscosity; $\dot{\gamma }$ is the shear strain rate; and *n*, *B*, *τ*^*^, and *T*_b_ are fitting parameters, which are shown in Table A1. These parameters are determined based on the temperature-dependent experimental data of complex viscosity following the Cox–Merz rule because the resins are all linear polymers. The shear viscosity can be calculated at an arbitrary temperature of *T*_r_.

**Table A1:** Cross model parameters of viscosity estimation of the polypropylene used.

	*n* (-)	*B* (Pa·s)	*τ** (Pa)	*T*_b_ (°C)
PP F-704NP	0.4212	8.486 × 10^−6^	12,360	9472.1

The order of the screw element’s code from the feed to the head of screw configurations in the catalog of SHIBAURA MACHINE [24] is presented in Table A2.

**Table A2:** The order of screw element’s code of screw configurations *.

	A	B	C		Residence Time Meas.
**Feeder**	-	-	-		-
1	CL-3	CL-3	CL-3		CL-3
2	SC-20/20	SC-20/20	SC-20/20		SC-20/20
3	SC-40/40	SC-40/40	SC-40/40		SC-40/40
4	SC-40/40	SC-40/40	SC-40/40		SC-40/40
5	SC-40/40	SC-40/40	SC-40/40		SC-40/40
6	SC-40/40	SC-40/40	SC-40/40		SC-40/40
7	SC-40/40	SC-40/40	SC-40/40		SC-40/40
8	SC-40/40	SC-40/40	SC-40/40		SC-40/40
9	SC-40/40	SC-40/40	SC-40/40		SC-40/40
10	SC-40/40	SC-40/40	SC-40/40		SC-40/40
11	SC-40/40	SC-40/40	SC-40/40		SC-40/40
12	SC-40/40	SC-40/40	SC-40/40		SC-40/40
13	SC-40/40	SC-40/40	SC-40/40		SC-40/40
14	SC-40/40	SC-40/40	SC-40/40		SC-40/40
15	SC-40/40	SC-40/40	SC-40/40		SC-40/40
16	SC-40/40	SC-40/40	SC-40/40		SC-40/40
17	SC-40/40	SC-40/40	SC-40/40		SC-40/40
18	SC-34/34	SC-34/34	SC-34/34		SC-34/34
19	SC-34/34	SC-34/34	SC-34/34		SC-34/34
20	SC-34/34	SC-34/34	SC-34/34		SC-34/34
21	SC-27/27	SC-27/27	KD-27/5R		SC-27/27
22	SC-27/27	SC-27/27	KD-27/5N		SC-27/27
23	SC-27/27	SC-27/27	KD-27/5L		SC-27/27
24	SC-27/27	SC-27/27	SC-10/20L		SC-27/27
25	SC-27/27	SC-27/27	SC-27/27		SC-27/27
26	SC-27/27	SC-27/27	SC-27/27		SC-27/27
27	SC-27/27	SC-27/27	SC-27/27		SC-27/27
28	SC-27/27	SC-27/27	SC-27/27		SC-27/27
29	SC-27/27	SC-27/27	KD-27/5R		SC-27/27
30	KD-27/5R	KD-27/5R	KD-27/5N		Open head
31	SC-27/27	KD-27/5N	KD-27/5L	←AE sensor here	-
32	SC-27/27	KD-27/5L	SC-10/20L		-
33	SC-27/27	SC-10/20L	SC-27/27		-
34	SC-27/27	SC-27/27	SC-27/27		-
35	SC-27/27	SC-27/27	SC-27/27		-
36	SC-27/27	SC-27/27	SC-27/27		-
37	SC-27/27	SC-27/27	SC-27/27		-
38	SC-27/27	SC-27/27	SC-27/27		-
39	SC-27/27	SC-27/27	SC-27/27		-
40	SC-27/27	SC-27/27	SC-27/27		-
41	SC-27/27	SC-27/27	SC-27/27		-
42	SC-27/27	SC-27/27	SC-27/27		-
43	SC-27/27	SC-27/27	SC-27/27		-
44	SC-27/27	SC-27/27	SC-27/27		-
45	SC-27/27	SC-27/27	SC-27/27		-
46	SC-27/27	KD-27/5R	SC-27/27		-
47	SC-27/27	KD-27/5N	SC-27/27		-
48	SC-27/27	KD-27/5L	SC-27/27		-
49	SC-27/27	SC-10/20L	SC-27/27		-
50	SC-34/34	SC-34/34	SC-34/34		-
51	SC-34/34	SC-34/34	SC-34/34		-
52	SC-34/34	SC-34/34	SC-34/34		-
53	SC-27/27	SC-27/27	SC-27/27		-
54	SC-20/20	SC-20/20	SC-20/20		-
55	SC-20/20	SC-20/20	SC-20/20		-
56	SC-20/20	SC-20/20	SC-20/20		-
57	SC-20/20	SC-20/20	SC-20/20		-
Head	-	-	-		-

* Screw element names do not reflect the dimensions of the screw element precisely. The exact dimension is a part of confidentiality. CL-3: 3 mm length collar, SC: Full-flight screw, KD: Kneading disk, R: Right-hand, N: Neutral, L: Left-hand configurations. For example, KD-27/5N is a neutral kneading disk of which the length and number of disks are 27 mm and 5, respectively. SC-10/20L is a full-flight reverse screw element of which length and pitch are 10 and 20, respectively.
